# Effects of traditional Chinese medicine polysaccharides on rheumatoid arthritis through gut microbiota modulation: a systematic review in animal models

**DOI:** 10.3389/fimmu.2026.1790486

**Published:** 2026-04-23

**Authors:** Suhai Qian, Yibo Jin, Chao Hu, Yuanyuan Luo, Runyu Chang, Jingyi Wu, Xinghong Ding

**Affiliations:** School of Basic Medical Sciences, Zhejiang Chinese Medical University, Hangzhou, China

**Keywords:** gut microbiota, gut-joint axis, immunomodulation, short-chain fatty acid, traditional Chinese medicine polysaccharide

## Abstract

**Background:**

Traditional Chinese medicine polysaccharides (TCMPs) are promising therapeutic candidates for rheumatoid arthritis (RA), known for their efficacy and low toxicity. Acting as potent gut microbiota modulators, TCMPs may alleviate RA by reshaping microbial communities and their metabolic outputs.

**Methods:**

This systematic review followed PRISMA guidelines. We searched PubMed, Web of Science, and Embase up to April 7, 2025, for relevant animal studies.

**Results:**

From an initial pool of 282 records, nine studies meeting the inclusion criteria were analyzed, involving polysaccharides from six single herbs and one compound formula. The results demonstrated that TCMPs significantly alleviated RA symptoms, including paw swelling and arthritis scores, and improved bone quality metrics. Regarding gut microbiota modulation, TCMPs induced changes in nine phyla (e.g., Patescibacteria, Desulfobacterota, Firmicutes) and 65 genera. At the genus level, 37 taxa (including *Dubosiella*, *Faecalibaculum*, *Bifidobacterium*) increased in abundance post-treatment, while 20 decreased. The results for 8 genera were inconsistent across studies. Notably, the abundance of *Lactobacillus* was reported to increase in four of the included studies. These microbial shifts correlated with reduced pro-inflammatory cytokines (e.g., IL-1β, TNF-α), improved RA clinical and bone parameters, and elevated levels of short-chain fatty acids (SCFAs), particularly butyrate and propionate. Correlation analyses identified Romboutsia and Lactobacillus as negatively associated with RA severity. Mechanistically, TCMPs enriched beneficial genera, enhanced SCFA production, suppressed NF-κB and JAK/STAT3 pathways, upregulated tight junction-related genes, and inhibited NLRP3 inflammasome activation via microbiota-derived metabolites, collectively forming a “microbiota–metabolite–host” regulatory network.

**Conclusion:**

This review demonstrates that TCMPs alleviate RA in animal models by modulating gut microbiota to enrich beneficial bacteria, suppress pathogens, enhance intestinal barrier function, and regulate immune homeostasis via the gut–joint axis, with SCFAs playing a pivotal role. Given the low methodological quality and high heterogeneity of included studies, future research should prioritize rigorous design, multi-omics integration, and clinical translation.

**Systematic review registration:**

https://www.crd.york.ac.uk/prospero/, identifier CRD420251053482.

## 1 Introduction

Rheumatoid arthritis (RA), a prevalent autoimmune disorder, affects approximately 1% of the global population (1). This condition is primarily characterized by inflammatory arthritis that typically involves the small joints of the hands and feet, and may lead to severe disability and mortality if not effectively controlled (2). Although current clinical treatments, including nonsteroidal anti-inflammatory drugs, glucocorticoids, and immunosuppressants, can alleviate symptoms, their long-term use is often associated with adverse effects such as gastrointestinal damage, hepatorenal dysfunction, and increased risk of infections, which significantly limit their clinical application (3–5). Consequently, the development of alternative therapeutic agents with high efficacy, low toxicity, and natural origins has emerged as a crucial research direction in the field of rheumatoid arthritis.

Traditional Chinese medicine (TCM) has garnered significant attention in the treatment of rheumatoid arthritis (RA) due to its notable therapeutic efficacy and relatively low toxicity (6). Among its bioactive components, polysaccharides exhibit multi-target regulatory effects in ameliorating RA. Studies have demonstrated that polysaccharides derived from TCM (TCMPs), such as those extracted from Clematis chinensis Osbeck, Anoectochilus roxburghii, and Astragalus, can effectively mitigate inflammation and synovial damage (7–9). Although polysaccharides, as macromolecular compounds, cannot be fully digested by the human body, they serve as potent modulators of gut microbiota by promoting the proliferation of beneficial intestinal bacteria while suppressing the growth of harmful microorganisms. This modulation enhances the production of short-chain fatty acids (SCFAs), improves intestinal mucosal barrier function, and activates specific signaling pathways (10). The gut microbiota, which has been shown to play a crucial role in host metabolism, has emerged as a novel therapeutic target for RA (11). Notably, gut microbial populations such as Firmicutes and Bacteroidetes can degrade polysaccharides through various carbohydrate-active enzymes (12, 13). Therefore, investigating the interaction between polysaccharides and gut microbiota to restore microbial homeostasis and modulate metabolic byproducts represents a critical research direction in evaluating the therapeutic potential of polysaccharides for RA.

This review comprehensively analyzes the existing literature regarding the effects of TCMPs on RA through modulation of gut microbiota and their metabolites. It systematically examines the therapeutic potential of TCMPs in animal models, their regulatory effects on gut microbiota composition and metabolic products, as well as the underlying molecular mechanisms. These findings provide a solid theoretical foundation for further clinical research on TCMPs as novel therapeutic agents for RA treatment.

## 2 Methods

### 2.1 Search strategy and terminology

To ensure methodological rigor and minimize bias, this study adhered to the Preferred Reporting Items for Systematic Reviews and Meta-Analyses (PRISMA) guidelines. The study protocol was registered on the PROSPERO international prospective register of systematic reviews (Registration No: CRD420251053482).

A comprehensive electronic search was conducted across Web of Science, PubMed, and Embase databases to identify all relevant experimental studies published before April 7, 2025. Additionally, reference lists of selected articles were manually screened to identify further pertinent literature.

The literature search was performed independently by two researchers: SH-Q designed the search strategy, while both SH-Q and YB-J executed the searches across all databases. Search terms included free-text keywords and Medical Subject Headings (MeSH) terms related to “polysaccharides,” “rheumatoid arthritis,” and “gut microbiota.” These terms were used in various combinations for title/abstract or full-text searches, for instance: polysaccharides AND rheumatoid arthritis AND gut microbiota (search strategy can be found in **Supplementary Table 1**).

Three independent investigators (SH-Q, YB-J, and C-H) performed preliminary data screening. Duplicate records were first removed based on PMID, title, author names, and publication year. Subsequently, articles were excluded through title/abstract screening by eliminating reviews, meta-analyses, letters, and clinical studies. Finally, full-text assessment and data extraction were conducted by two researchers (SH-Q and C-H). Any discrepancies were resolved through consensus discussion or consultation with a third expert. For unavailable full-text articles, corresponding authors were contacted via email for document requests.

### 2.2 Inclusion and exclusion criteria

The inclusion criteria comprised: (1) animal studies investigating rheumatoid arthritis; (2) interventions utilizing polysaccharides derived from traditional Chinese medicine (TCM), which were categorized by source as either single-herb polysaccharides or compound formula polysaccharides, with the stipulation that all involved TCM must be officially documented in the *Chinese Pharmacopoeia* issued by the National Medical Products Administration and the National Health Commission - only pharmacopoeia-listed species qualify as legally recognized TCM; (3) outcome measures encompassing both rheumatoid arthritis assessment parameters and gut microbiota analysis; (4) study designs restricted to randomized controlled trials (RCTs).

The exclusion criteria were as follows: (1) animal models of RA complications; (2) studies with flawed experimental designs (e.g., using non-RA animal models, failing to clearly describe modeling criteria or procedures, or containing major methodological flaws that compromise result interpretation); (3) interventions involving non-TCMPs, such as polysaccharides derived from fungi or bacteria, non-polysaccharide components, or mixtures of TCMPs with other ingredients; (4) review articles, letters to the editor, master’s or doctoral theses, articles with obvious errors (including but not limited to inconsistencies between Chinese and English text, confusion in group descriptions, or incomplete experimental design descriptions), and unpublished data.

### 2.3 Study selection and data extraction

Two independent investigators (SH-Q and YB-J) performed data extraction and compiled summary tables. The extracted data were systematically organized into tables or figures according to content analysis and review structure. Any discrepancies between SH-Q and YB-J were resolved through discussion with a third researcher (XH-D) to reach consensus.

### 2.4 Critical appraisal of included studies

To enhance the validity and reliability of this systematic review, we employed the Systematic Review Centre for Laboratory Animal Experimentation’s Risk of Bias tool (SYRCLE’s RoB tool) to scientifically evaluate methodological quality of included studies. Adapted from the Cochrane Risk of Bias tool for randomized controlled trials, SYRCLE’s RoB tool specifically assesses internal validity in animal studies (14). The instrument evaluates six domains through ten items: (1) selection bias (sequence generation, baseline characteristics, allocation concealment); (2) performance bias (random housing and blinding); (3) detection bias (random outcome assessment and blinding); (4) attrition bias (incomplete outcome data); (5) reporting bias (selective outcome reporting); and (6) other potential bias sources. Each item was judged using “+” (low risk), “-” (high risk), or “?” (unclear risk due to insufficient information), with “+” judgments scoring 1 point and other ratings scoring 0. Two independent reviewers (SH-Q and C-H) conducted assessments, resolving discrepancies through discussion with the corresponding author to reach consensus.

## 3 Results

### 3.1 Search results

**Figure 1:**
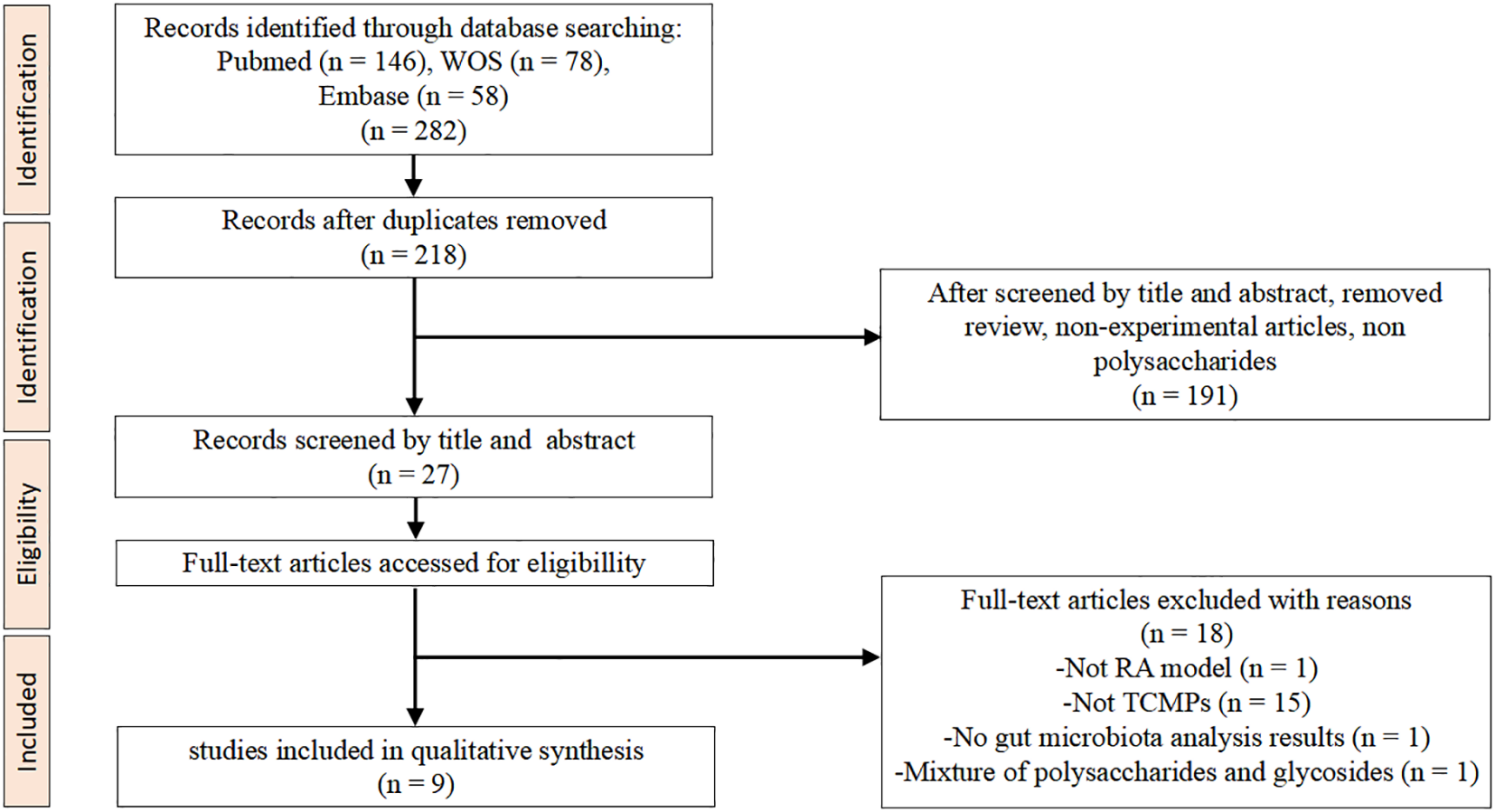
Flowchart of the process for selecting studies. Illustrates the detailed data screening process.

The systematic literature search initially identified a total of 282 articles across all databases, which were reduced to 218 after duplicate removal. Preliminary screening of titles and abstracts based on the inclusion and exclusion criteria resulted in 27 potentially eligible articles. Following comprehensive full-text evaluation, 18 articles were excluded for the following reasons: utilization of non-RA animal models, interventions not involving TCMPs, absence of gut microbiota analysis data, or inclusion of polysaccharide-glycoside mixtures. The final analysis included nine studies that satisfied all selection criteria (**Figure 1**). This rigorous screening process ensured the inclusion of only the most methodologically sound and relevant studies for our systematic review.

### 3.2 Quality assessment of included studies

**Figure 2:**
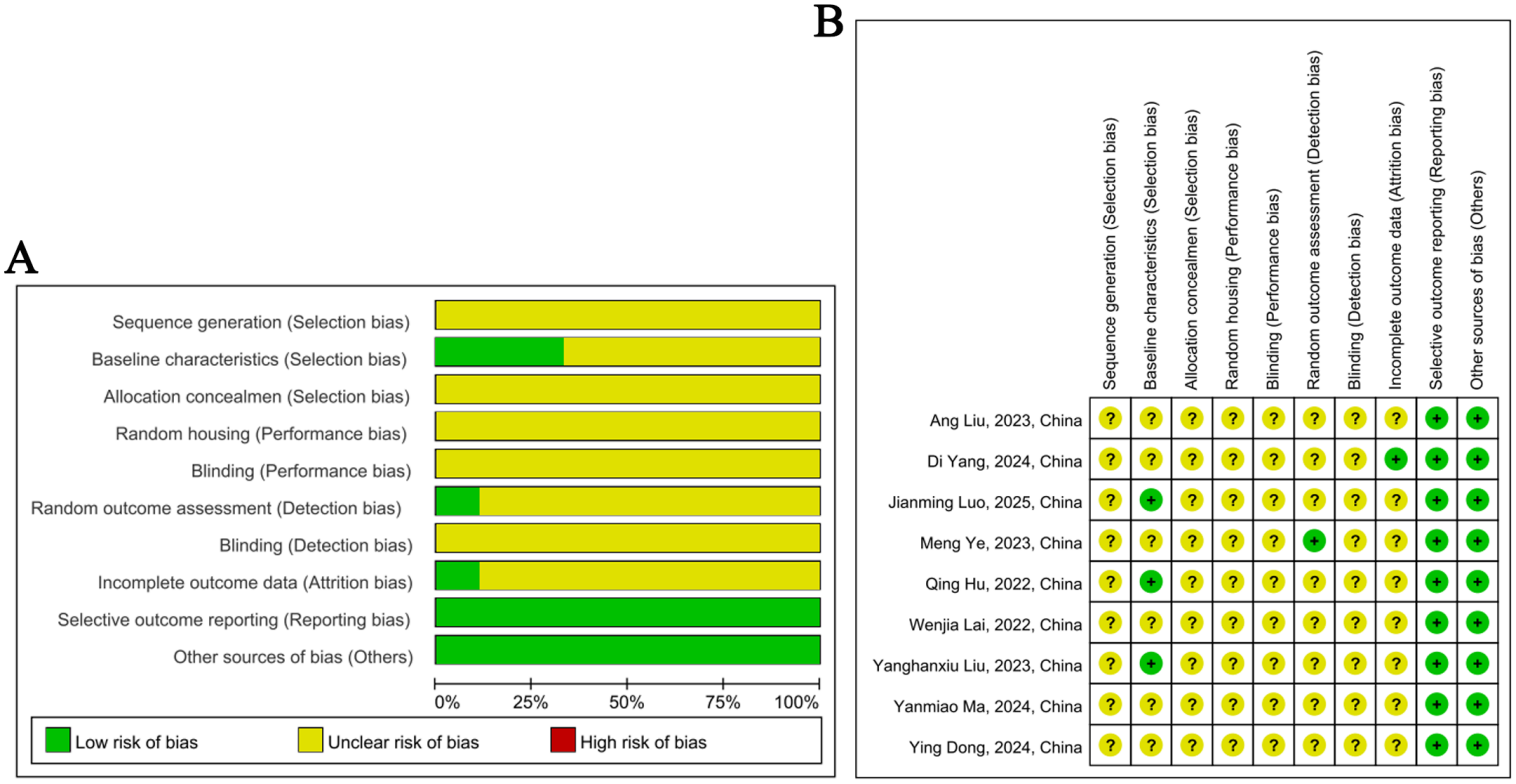
Results of risk of bias assessment. **(A)** Risk of bias graph; **(B)** Risk of bias summary; +, low risk of bias; -, high risk of bias; ?, unclear.

We assessed the quality of all included studies, totaling nine studies (15–23). Each study scored between 2 and 3, indicating low overall study quality. Specifically, five studies scored 3 points (15, 18, 19, 22, 23), while four studies scored 2 points (16, 17, 20, 21) (detailed bias assessment scores are provided in **Supplementary Table 2**). The majority of the studies merely mentioned “randomization” without specifying the randomization method or clarifying whether allocation concealment was implemented, leading to an “unclear” rating in these domains. Only three studies described “random allocation based on body weight,” which was considered indicative of similar baseline characteristics (15, 18, 23). None of the studies mentioned random cage allocation, and no information was provided regarding the blinding of researchers during intervention implementation, potentially introducing performance bias, which was consequently rated as “unclear.” Most studies lacked adequate information regarding detection bias related to blinding of outcome evaluators, so they were rated as “unclear”. A single study mentioned “random selection of three fecal samples per group for gut microbiota analysis,” which was rated as “low risk” for random outcome assessment (19), whereas the remaining studies did not report random outcome selection. The completeness of reported data was unclear in most studies, as some failed to specify the number of animals included in the results, while others reported only partial data without accounting for the remaining animals. Only one study maintained consistent animal numbers between the methods and results sections and reported all experimental data, warranting a “low risk” rating (22). No other high-risk sources of bias were identified in the included studies. A detailed risk-of-bias assessment using the SYRCLE’s RoB tool is presented in **Figure 2**. In summary, the methodological quality of all studies requires improvement due to unclear random sequence generation, unclear allocation concealment, unclear random outcome assessment, lack of blinding, and insufficient reporting of data completeness.

### 3.3 Study overview

#### 3.3.1 TCMPs

Traditional Chinese Medicine (TCM) represents one of the world’s oldest medical systems, with most traditional herbal substances derived from botanical sources. Polysaccharides, as the primary constituents of medicinal herbs, exhibit significant bioactivities, including antitumor, antioxidant, antidiabetic, radioprotective, antiviral, hypolipidemic, and immunomodulatory effects, while demonstrating relatively low toxicity (24). Our systematic review analyzed nine studies involving polysaccharides from six medicinal herbs and one compound formula (**Table 1**). Among them, two polysaccharides were derived from Angelica sinensis (15, 23), and two were extracted from Lycium barbarum (16, 18). Regarding extraction methods, eight studies employed water extraction followed by alcohol precipitation (15, 17–23), while only one study did not specify the extraction process as it utilized commercially available polysaccharides (16). Given that monosaccharide composition serves as the foundation for the structural and functional diversity of polysaccharides (25), we summarized these data in **Table 1**. Additionally, since many studies reported molecular weight and polysaccharide composition, we also compiled these details, revealing considerable variation in molecular weight and structural composition across different polysaccharide sources (**Table 1**).

**Table 1:** Plant sources and composition of TCMPs.

Refs.	TCMPs	Source	Part used	Monosaccharide composition and ratio (or molar ratio)	Molecular weight	Composition (%)
Hu et al., 2022 (15)	Angelica sinensis polysaccharide (ASP)	*Angelica sinensis* (Oliv.) Diels	root	glucose, mannose, galactose, rhamnose, arabinose, and xylose (Exact ratio unknown).	NA	polysaccharide: 80
Lai et al., 2022 (16)	Lycium barbarum polysaccharide (LBP)	*Lycium barbarum* L.	fruit	Mannose: Ribose: Rhamnose: Glucuronic acid: Galacturonic acid: Glucose: Galactose: Xylose: Arabinose=6:1:2:1:9:38:10:12:21	NA	NA
Liu et al., 2023 (17)	Acanthopanax senticosus polysaccharide (ASPS)	*Eleutherococcus senticosus* (Rupr. & Maxim.) Maxim.	root	D-galactose (Gal): D-glucose (Glc): L-arabinose (Ara): D-rhamnose (Rha): D-galacturonic acid (GalA): D-mannose (Man): D-glucuronic acid (GlcA) = 11:9:7:5:5:1:1	1.65 × 10^5^ Da	NA
Liu et al., 2023 (18)	Lycium barbarum polysaccharide (LBP)	*Lycium barbarum* L.	fruit	glucose, arabinose, galactose, mannose, xylose, rhamnose, fucose, galacturonic acid, glucuronic acid (Exact ratio unknown).	NA	NA
Ye et al., 2023 (19)	Notopterygium incisum polysaccharide (NIP)	*Notopterygium incisum* K.C.Ting ex H.T.Chang	root	arabinose, galactose, glucose, and galacturonic acid = 1:2.01:3.30:1.12	2.34×10^6^ Da	the neutral sugar content (72.87 ± 1.10), the uronic acid content (23.55 ± 4.27), the protein content (0.29 ± 0.40).
Dong et al., 2024 (20)	Dianbaizhu polysaccharide (DBZP)	*Gaultheria leucocarpa var. yunnanensis* (Franch.) T.Z. Hsu & R.C. Fang	aerial parts	mannose, ribose, rhamnose, glucuronic acid , galacturonic acid, glucose, galactose, xylose, arabinose = 6.63: 1.33: 4.53: 2.95: 32.29: 13.78: 22.97: 3.94: 11.59	> 2457 kDa	moisture content (8.06 ± 0.09), total carbohydrates content (33.87 ± 0.29) , total protein content (1.70 ± 0.02), uronic acid content (28.35 ± 0.31), total phenol content 168.73 ± 7.77 mg GAE/g
Ma et al., 2024 (21)	Ephedra sinica polysaccharide (ESP)	*Ephedra sinica* Stapf	stem	D-Mannose, Glucosamine, Ribose, L-Rhamnose monohydrate, D-Glucuronic acid, D-Galacturonic acid, Glucose, Galactose, D-Xylose, Arabinose, Fucose = 2828.57 : 460 : 1057.14 : 2674.29 : 3334.29 : 11214.29 : 33622.86 : 5500 : 651.43 : 10140 : 102.86	NA	NA
Yang et al., 2024 (22)	polysaccharide (PS) in Wu-tou decoction (WTD)	Aconiti Radix Preparata, Ephedrae Herba, Glycyrrhiza Radix, Paeoniae Radix Alba, and Astragali Radix.	root, stem, root, root, root	NA	NA	polysaccharide: 78.89±0.89, it also contains Calycosin, Liquiritin, 5-OH liquiritin, Hypaconine, Liquiritin apiosid (specific proportion unknown).
Luo et al., 2025 (23)	Purification of ASP-2pb from polysaccharides of Angelica sinensis (ASP)	*Angelica sinensis* (Oliv.) Diels	root	arabinose, galactose, and galacturonic acid = 30.36: 62.08: 7.57	92.02 kDa	total carbohydrate content: 90.29, trace protein content: below 0.01

Abbreviations: TCMPs, traditional Chinese medicine polysaccharides.

#### 3.3.2 Experimental summary

##### 3.3.2.1 Basic characteristics of the included studies

The key characteristics of the nine included studies are summarized in **Table 2**. These studies were published between 2022 and 2025, with two studies in 2022, three each in 2023 and 2024, and one in 2025. All studies were conducted in China.

**Table 2:** Basic characteristics of studies.

First author, year of publication	TCMPs	Animal Type, sex (F/M), weight (g), age (week)	RA modeling methods	Sample size	Interventions model	Interventions positive (mg kg^- 1^/d)	Interventions treatment (mg kg^- 1^/d)	Interventions duration (d)
Hu et al., 2022 (15)	ASP	Wistar rats, F, NA, 8 weeks old	The model was established by a double immunization: bovine type II collagen was emulsified with the same volume of IFA, and the emulsion was injected twice subcutaneously from the root of the rat tail.	T: 40 N: 8	Gavage of sterile water	NA	Gavage of ASP sterile saline solution (200,400, 800)	35
Lai et al., 2022 (16)	LBP	Wistar rats, F, NA, 8 weeks old	The model was established by a double immunization: an equal volume of bovine type II collagen was mixed and emulsified with IFA, and the rats were immunized with two subcutaneous injections.	T: 24 N: 8	Gavage of equal volume of sterilized distilled water	NA	Gavage of LBP sterile saline solution (400)	42
Liu et al., 2023 (17)	ASPS	DBA/1 mice, M, NA, 8 weeks old	The model was established by a double immunization: for the first immunization, bovine type II collagen was emulsified with equal volume CFA, and the emulsion was injected subcutaneously from the tail of the mice; for a second immunization, IFA instead of CFA was mixed with type II bovine collagen and an emulsion was injected subcutaneously from the tail of the mice.	T: 32 N: 8	Gavage of the same volume of blank solvent water	NA	Gavage of ASPS sterile saline solution (300)	51
Liu et al., 2023 (18)	LBP	Wistar rats, F, NA, 7–8 weeks old	The model was established by a double immunization: an equal volume of bovine type II collagen was mixed and emulsified with IFA, and the rats were immunized with two subcutaneous injections.	T: 30 N: 6	Gavage of the same amount of distilled water	NA	Gavage of LBP sterile saline solution (400)	22
Ye et al., 2023 (19)	NIP	SD rats, M, 160–180g, 6–8 weeks old	CFA was subcutaneously injected into the right rear toe of rats for one time.	T: 60 N: 10	Gavage of equal amount of distilled water	Gavage of dexamethasone solution (9.4)	Gavage of NIP sterile saline solution (200, 400, 800)	14
Dong et al., 2024 (20)	DBZP	DBA/1 J mice, M, 20 ± 2, NA	The model was established by a double immunization: for the first immunization, bovine type II collagen was emulsified with equal volume CFA, and the emulsion was injected subcutaneously from the tail of the mice; for a second immunization, IFA instead of CFA was mixed with type II bovine collagen and an emulsion was injected subcutaneously from the tail of the mice.	T: 50 N: 10	Gavage of distilled water	Gavage of dexamethasone solution (1)	Gavage of DBZP sterile saline solution (440, 1760)	28
Ma et al., 2024 (21)	ESP	C57BL/6J mice, NA, NA, NA	The model was established by a double immunization: type II collagen and CFA were fully emulsified at a 1:1 ratio, and then the emulsion was injected into the tail, back, and left hind paw of the mice, and a week later the emulsion was injected into the same sites to strengthen immunization.	NA;NA	Gavage of saline	Gavage of methotrexate solution (9)	Gavage of ESP sterile saline solution (200, 400)	21
Yang et al., 2024 (22)	PS in WTD	SD rats, M, 180–220g, NA	CFA was given to each rat on the left hind foot, and AIA model was successfully established two weeks later.	T: 42 N: 6	Gavage of the same amount of saline	Gavage of WTD (9.8)	Gavage of PS sterile saline solution (9.8)	study of intestinal microflora: 30; PK study: 21
Luo et al., 2025 (23)	ASP-2pb	Wistar rats, F, NA, 7–8 weeks old	The model was established by a double immunization: bovine type II collagen was emulsified with the same volume of IFA, and the emulsion was injected twice subcutaneously from the root of the rat tail.	T: 40 N: 6	Gavage of the same amount of sterile distilled water	NA	Gavage of ASP-2pb sterile saline solution (400)	39

Abbreviations: ASP, Angelica sinensis polysaccharide; ASPS, Acanthopanax senticosus polysaccharide; CFA, complete Freund’s adjuvant; DBZP, Dianbaizhu polysaccharide; ESP, Ephedra sinica polysaccharide; GF, germ-free; IFA, incomplete Freund's adjuvant; KO, knockout; LBP, Lycium barbarum polysaccharide; NIP, Notopterygium incisum polysaccharide; PBS, phosphate-buffered saline; PS, polysaccharide; TCMPs, traditional Chinese medicine polysaccharides; TLR2, Toll-like receptor 2; WTD, Wu-tou decoction.

The animal models used in these studies were exclusively rats or mice (**Table 2**). Among them, three studies employed mice (17, 20, 21), including two utilizing DBA/1 mice (17, 20) and one using C57BL/6J mice (21). The remaining six studies used rats (15, 16, 18, 19, 22, 23), with four involving Wistar rats (15, 16, 18, 23) and two utilizing SD rats (19, 22). The sex distribution was clearly specified in rat models, with Wistar rats being exclusively female and SD rats exclusively male. Since many studies did not report the initial body weight of the animals, this parameter was not summarized. Regarding age, six studies reported animals within the 6–8-week range (15–19, 23), while three studies did not provide this information.

For RA model induction, seven studies adopted a dual immunization protocol (15–18, 20, 21, 23), whereas two studies employed a single injection of CFA (19, 22). Across the studies, sterile water, distilled water, blank solvent water, or normal saline were used as control interventions. Positive control groups were treated with dexamethasone, methotrexate, or Wu-tou decoction (WTD). However, five studies did not report any positive control interventions. The dosages of polysaccharides varied across studies, with specific values detailed in **Table 2**. The duration of interventions ranged from a minimum of 14 days to a maximum of 51 days.

##### 3.3.2.2 Microbiome assessment methods

All nine included studies analyzed fecal or intestinal samples. Among them, five studies utilized fecal samples (17, 19–21, 23), one employed colonic contents (22), and three used cecal contents (15, 16, 18). Five studies explicitly stated that samples were stored at –80 °C (15, 16, 18–20), while one study reported that cecal contents and joint tissues were rapidly frozen in liquid nitrogen and maintained at –80 °C, though the storage conditions for fecal samples used in gut microbiota analysis were not specified (23). The remaining studies did not report sample preservation conditions.

**Figure 3:**
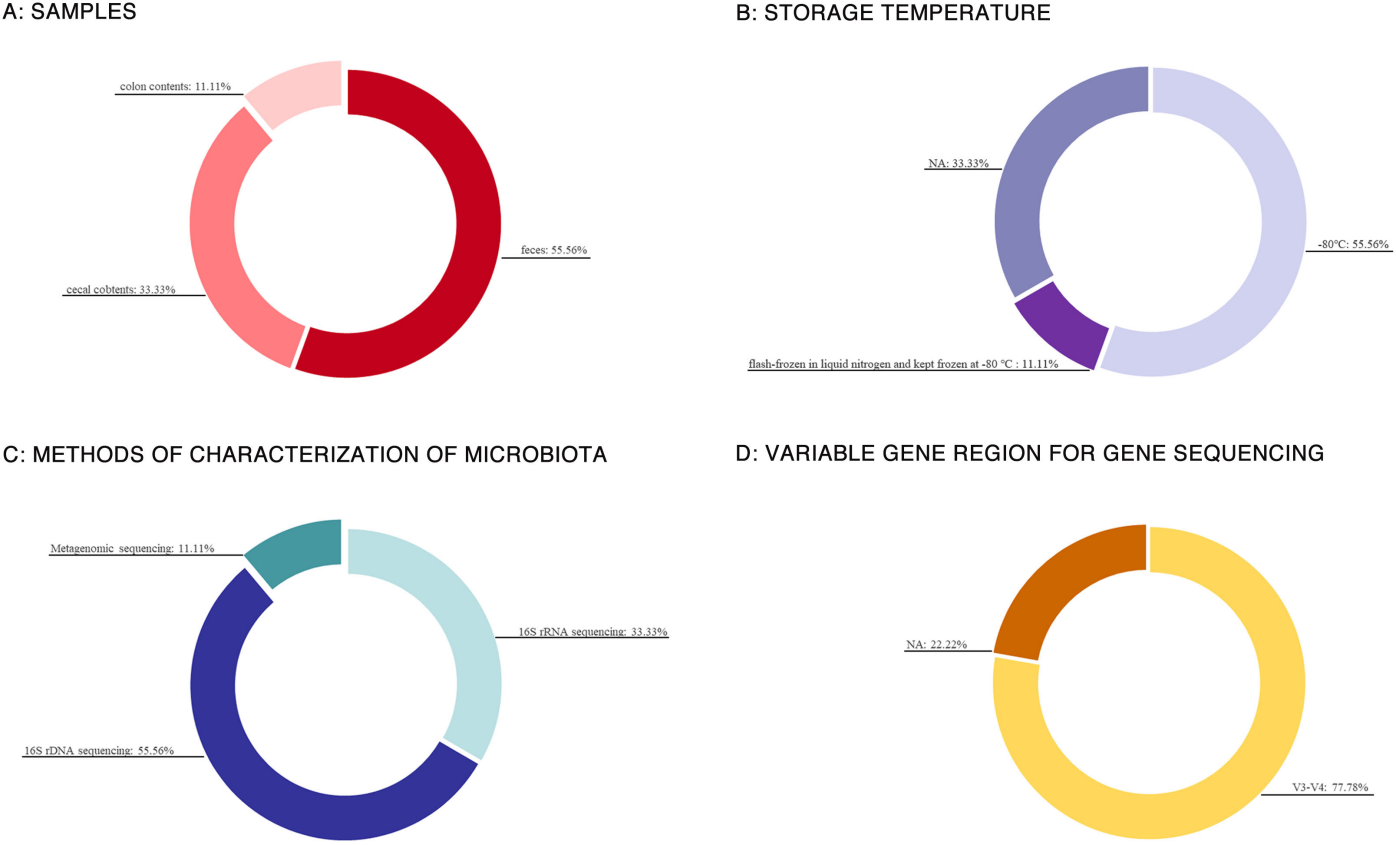
Method of microbiome assessment. **(A)** Samples, **(B)** Storage temperature, **(C)** Methods of characterization microbiota, **(D)** Variable gene region for gene sequencing.

Regarding microbial profiling, 16S rRNA sequencing was applied in three studies (17, 20, 22), 16S rDNA sequencing in five studies (15, 16, 18, 19, 23), and metagenomic sequencing in one study (21). Among the studies employing gene sequencing, the V3-V4 hypervariable region was universally targeted where specified (15, 16, 18–20, 22, 23) (**Figure 3**).

#### 3.3.3 Therapeutic effects of TCMPs on RA

Rheumatoid arthritis (RA), the most prevalent systemic inflammatory arthritis, is diagnostically characterized by clinically detectable swelling in at least one joint (26). Arthritis scoring systems facilitate the quantification of complex clinical manifestations through standardized metrics, enabling objective assessment of disease activity (27). Among the nine included studies, all incorporated paw/joint swelling indices, with six further evaluating histopathological arthritis scores (16–18, 20, 21, 23). Quantitative assessment of bone microstructure and quality is critical in RA management (28). Parameters such as bone volume (BV), bone surface area (BS), BV/total volume (TV), BS/BV, BV/TV, trabecular thickness (Tb.Th), trabecular number (Tb.N), bone mineral density (BMD), and structure model index (SMI) not only guide personalized anti-inflammatory and anti-osteoporotic therapies but also serve as objective predictors of fracture risk and prognostic evaluation. Our analysis extracted these bone-related metrics across studies. Given that most animal models employed collagen-induced arthritis (CIA), serum levels of type II collagen antibodies and their subtypes hold pathological significance in RA pathogenesis. As summarized in **Table 3**, TCMPs demonstrated marked efficacy in attenuating RA-specific indices, reducing paw swelling, and ameliorating bone deterioration.

**Table 3:** The regulation of gut microbiota and the improvement of RA by TCMPs.

Refs.	TCMPs	Improvement of RA	Oxidative stress; Inflammatory levels	Gut microbiota diversity (α-diversity; β-diversity)	F/B	SCFAs	Genes and proteins in related pathways
Hu et al., 2022 (15)	ASP	paw swelling↓, CII-IgG↓, CII-IgG1↓, CII-IgG2a↓, CII-IgG2b↓	NA; TNF-α↓	NA; PLS-DA: the apparent distance among the different groups was far.	NA	NA	DEGs and mRNA in Colon: Adcy4↑, Cldn5↑, Myl9↑, Slit3↑, Rgs18↑
Lai et al., 2022 (16)	LBP	paw swelling↓, arthritis score↓	NA; IL-1α↓, IL-1β↓, IL-12↓, IL-17A↓, IL-10↑	NA; PLS-DA: the apparent distance among the different groups was far.	NA	NA	methylome in colonic epithelial cells: FPKM↓, methylation level↑ (10 genes including Dpep3, Gstm6, Slc27a2, Col11a2, Sycp2, SNORA22, Tnni1, Gpnmb, Mypn and Acsl6 )
Liu et al., 2023 (17)	ASPS	paw thickness↓, arthritis score↓, BV/TV↑, BV↑, BS↑, MMI↑, Tb.N↑, Tb.Th↑, total porosity↓, HSS↓, OARSI scores↓	NA; IL - 1β↓, TNF - α↓, F4/80↓, caspase‐1↓	shannon's diversity↑, pielou's evenness↑; PCoA based on Bray–Curtis distance: significant differences in gut microbial communities between groups.	↑	NA	Gene: NLRP3↓; protein: ASC↑
Liu et al., 2023 (18)	LBP	paw swelling↓, arthritis score↓	NA; IL-1α↓, IL-1β↓, IL-6↓, TNF-α↓	Chao index: NS, Shannon index: NS, Ace index: NS; PLS-DA: the apparent distance among the different groups was far.	NA	acetic acid↑, propionic acid↑, butyric acid↑, isobutyric acid↑, valeric acid↑, isovaleric acid inside↑	NA
Ye et al., 2023 (19)	NIP	foot swelling↓	MDA↓, SOD↑; TNF-α↓, IL-6↓, IL-17↓, IL-1β↓, IL-10: NS	Shannon index↑, Chao1↑; NA	↓	butyrate↑	proteins in spleen: p-p65/p65↓, p-STAT3/STAT3↓
Dong et al., 2024 (20)	DBZP	paw swelling↓, arthritis score↓, BV/TV↑, Tb.Th↑, BV↑, BS↑, BS/BV↓, BMD↑, SMI↓	NA;NA	Chao index: NS, Shannon index: NS, Ace index: NS; hierarchical cluster analysis: the groups were distributed in different layers, PCoA and NMDS: the groups were distributed in different areas.	NA	NA	NA
Ma et al., 2024 (21)	ESP	joint swelling↓, collagenous fiber↑, Mankin cartilage tissue score↓, OARSI joint pathology score↓	NA; IL-1β↓, IL-6↓	Chao1 index: NS, Shannon index: NS, Simpson index: NS; PCA, PCoA: some areas are separated. (Although the α diversity described in the study was improved, the Figure involved did not show a significant difference.)	NA	propionic acid↓, butyric acid↑	proteins in synovial: HDAC1↓, HDAC2↓, TLR4↓, MyD88↓, TRAF6↓, p-p65/p65↓, protetin in colon: ZO-1↑
Yang et al., 2024 (22)	PS in WTD	ankle swelling↓	NA; TNF-α↓, IL-6↓	NA; PCoA: there were significant differences among the groups.	↓	NA	NA
Luo et al., 2025 (23)	ASP-2pb	hind paw diameter↓, arthritis index score↓	NA; IL-1β↓, IL-1α, IL-6↓, TNF-α↓, IL-10↑	Ace index: NS, chao index: NS, Shannon index↑, Simpson index↓; PLS-DA: the apparent distance among the different groups was far.	↓	NA	NA

Abbreviations: ASP, Angelica sinensis polysaccharide; ASPS, Acanthopanax senticosus polysaccharide; BMD, bone mineral density; BS, bone surface area; BV, bone volume; DBZP, Dianbaizhu polysaccharide; DEGs, differentially expressed genes; ESP, Ephedra sinica polysaccharide; F/B, Firmicutes/Bacteroidota; HSS, histological synovitis scores; LBP, Lycium barbarum polysaccharide; MDA, malondialdehyde; MMI, polar moment of inertia; NIP, Notopterygium incisum polysaccharide; NMDS, non-metric multidimensional scaling analysis; PCoA, principal coordinate analysis; PLS-DA, partial least squares discriminant analysis; PS, polysaccharide; SAM, S-adenosyl methionine; SMI, structure model index; SOD, superoxide dismutase; Tb.N, trabecular number; Tb.Th, trabecular thickness; TCMPs, traditional Chinese medicine polysaccharides; TV, total volume; WTD, Wu-tou decoction.

Inflammation and oxidative stress form a vicious cycle that synergistically promotes RA progression (29). Our systematic analysis of the nine included studies revealed consistent modulatory effects of polysaccharides on key inflammatory and oxidative stress markers (**Table 3**). The evaluated inflammatory cytokines, including TNF-α, IL-1α, IL-1β, IL-12, IL-17A, and IL-17, demonstrated significant reduction following polysaccharide treatment, while anti-inflammatory IL-10 levels were elevated, indicating effective inflammatory modulation. Mechanistic investigations identified NF-κB overexpression as critically involved in RA pathogenesis, where its activation triggers cytokine release and upregulates matrix metalloproteinase genes, thereby accelerating extracellular matrix and cartilage degradation. Concurrently, IL-6-mediated JAK/STAT3 pathway activation in synovial cells was shown to exacerbate bone and cartilage damage (19). Notably, Notopterygium incisum polysaccharide (NIP) effectively suppressed both NF-κB and JAK/STAT3 signaling pathways (19). Another study also focused on the inflammation-related pathway of NF-κB, proposing that Ephedra sinica polysaccharide (ESP) reduces the levels of TLR4, MyD88, and TRAF6 in synovial membranes, inhibits the expression of p65 and phosphorylation of pp65 in the NF-κB signaling pathway, and blocks histone deacetylase (HDAC1 and HDAC2) signaling (21). Regarding oxidative stress parameters, the sole study evaluating malondialdehyde (MDA) and superoxide dismutase (SOD) activity confirmed the anticipated attenuation of oxidative stress markers under polysaccharide treatment (19). These collective findings demonstrate that TCMPs exert therapeutic effects through coordinated downregulation of pro-inflammatory cytokines, inhibition of NF-κB and JAK/STAT3 pathways, modulation of oxidative stress markers, and upregulation of anti-inflammatory mediators.

#### 3.3.4 Modulation of gut microbiota by TCMPs

Both clinical and animal studies have consistently demonstrated dysbiosis or compositional alterations in gut microbiota among RA patients and animal models (30, 31). Regarding α-diversity and β-diversity, a systematic review of 92 observational studies revealed significantly reduced α-diversity indices in RA patients (32), while another study reported no significant differences in α-diversity between healthy controls (HC) and RA groups, despite notable β-diversity distinctions (33). Among the nine included studies, six investigated α-diversity, with two demonstrating significant increases following polysaccharide treatment (17, 19), three showing no significant differences (18, 20, 21), and one reporting mixed results across different α-diversity indices (23). β-diversity was examined in eight studies, all of which reported significant alterations after TCMPs intervention (15–18, 20–23) (**Table 3**). Collectively, these findings indicate that TCMPs modulate both α-diversity and β-diversity of gut microbiota, though such analyses cannot discern specific phylum-level or genus-level differences, necessitating further compositional analysis.

At the phylum level, TCMPs treatment induced changes in nine bacterial phyla associated with RA: Patescibacteria, Desulfobacterota, Fusobacteria, Firmicutes, Proteobacteria, Actinobacteria, Deferribacterota, Bacteroidota (Bacteroidetes), and Tenericutes (**Supplementary Table 3**). Notably, Patescibacteria (4 studies), Firmicutes (3 studies), Actinobacteria (3 studies), and Bacteroidota (3 studies) were the most frequently studied, though their responses varied across studies. Three studies reported increased Patescibacteria abundance post-treatment, while two showed decreased Firmicutes levels. For Actinobacteria and Bacteroidota, two studies each documented post-treatment increases. The Firmicutes/Bacteroidota (F/B) ratio was evaluated in four studies, with three reporting decreased ratios and one showing an increase following polysaccharide treatment (**Table 3**).

**Figure 4:**
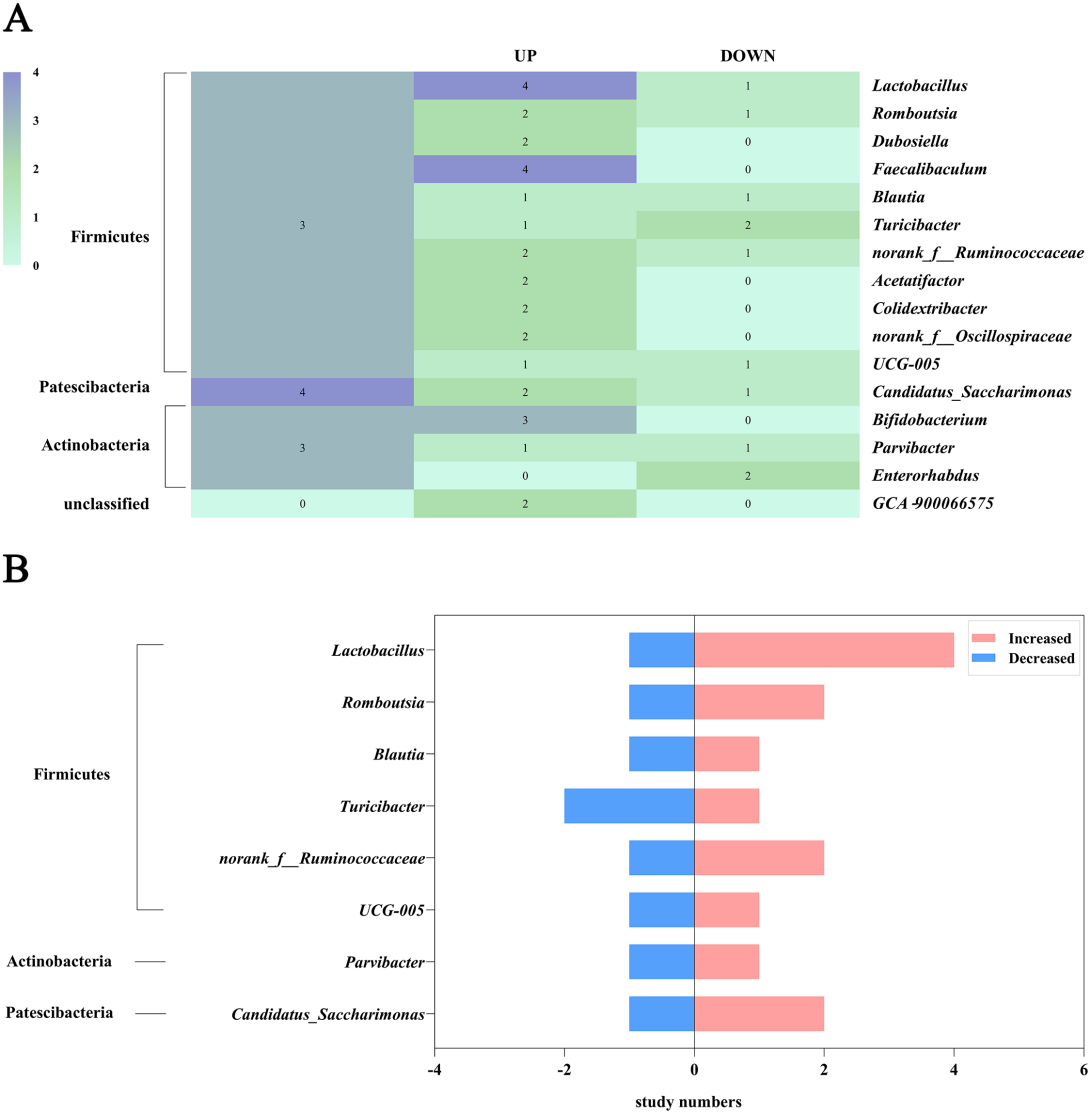
Heat map of intestinal phylum and genus. **(A)** Number of studies in which bacterial phyla and genus abundance increased or decreased after polysaccharide treatment. **(B)** Number of studies in which bacterial genus abundance increased and decreased after polysaccharide treatment.

Genus-level analysis across the nine studies identified 65 differentially abundant taxa after TCMPs intervention. Polysaccharide treatment significantly increased 37 genera, including *Dubosiella*, *Faecalibaculum*, *Acetatifactor*, *Bacteroides*, and *Bifidobacterium*, while decreasing 20 others such as *Enterorhabdus*, *Roseburia*, *Streptococcus*, and *Prevotella_2* (**Supplementary Table 4**). Sixteen genera were consistently reported in at least two studies (**Figure 4A**), including Firmicutes members (*Lactobacillus*, *Romboutsia*, *Dubosiella*, *Faecalibaculum*, *Blautia*, *Turicibacter*, *norank_f:Ruminococcaceae*, *Acetatifactor*, *Colidextribacter*, *norank_f:Oscillospiraceae*, *UCG-005*), Actinobacteria (*Bifidobacterium*, *Parvibacter*, *Enterorhabdus*), Patescibacteria (*Candidatus_Saccharimonas*), and unclassified GCA‐900066575. Among these, *Dubosiella*, *Faecalibaculum*, *Acetatifactor*, *Colidextribacter*, *norank_f:Oscillospiraceae*, *Bifidobacterium*, and *GCA‐900066575* showed consistent increases, while *Enterorhabdus* decreased post-treatment. Eight genera exhibited conflicting results across studies (**Figure 4B**), with *Lactobacillus* being the most frequently investigated (5 studies). While four studies reported increased *Lactobacillus* abundance after TCMPs intervention, one study demonstrated the opposite trend.

#### 3.3.5 Effects of TCMPs on gut microbiota-derived metabolites

Seven of the nine included studies investigated alterations in gut microbiota-related metabolites. Lai et al. proposed that the therapeutic mechanism of Lycium barbarum polysaccharide (LBP) in RA may involve remodeling gut microbiota to increase S-adenosylmethionine (SAM) levels, subsequently inducing DNA hypermethylation to suppress RA-associated gene expression (16). Liu et al. demonstrated that Acanthopanax senticosus polysaccharide (ASPS) treatment elevated γ-glutamylcysteine (GGC) concentrations in CIA mice, suggesting GGC as a microbial metabolite mediating ASPS’s anti-arthritic effects (17). Metabolomic analysis of cecal contents by Liu et al. revealed that LBP intervention significantly modulated intestinal metabolites, increasing nine metabolites (O-desmethylangolensin, 3-hydroxydodecanedioic acid, N-formyl-L-methionine, suberic acid, (S)-oleuropeic acid, prolyl-histidine, 13,14-dihydro PGF-1α, (R)-pelletierine, and 3-(acetyloxy)-2-hydroxypropyl icosanoate) while decreasing creatinine levels, identifying these ten metabolites as potentially critical to LBP’s therapeutic effects (18). Dong et al. analyzed fecal and urinary metabolomes, finding that Dianbaizhu polysaccharide (DBZP) treatment reversed RA-induced changes in 21 elevated and 3 reduced fecal metabolites, with 11β-hydroxytestosterone showing significant correlations with pathological and bone parameters (20). Urinary metabolites including nudifloramide, 3-methoxytyrosine, pyroglutamylglycine, cytidine, and 3-hydroxyanthranilic acid decreased post-treatment, while N-Acetyl-Lglutamate 5-semialdehyde, N(6)-methyllysine, tyrosyl-isoleucine, citrulline, D-alanyl-D-alanine and N-acetyldopamine increased, demonstrating close associations with gut microbiota and bone parameters (20). Another fecal metabolomics study reported elevated L-tyrosine and sn-glycero-3-phosphocholine alongside reduced hexadecanoic acid, octadecanoic acid, N-oleoyl taurine, and N-palmitoyl taurine following ESP treatment (21). The 2025 study highlighted metabolites produced by specific bacteria after ASP-2pb intervention as key to alleviating RA, with the abundance of 98 of these metabolites elevated after RA modeling but decreasing to normal levels after ASP-2pb intervention (23). In addition, choline, myristoleic acid, cuminaldehyde, 4-deoxypyridoxine, 10-(2,3-dihydroxypropoxy)-10-oxodecanoic acid, Val Pro Gln, linalool oxide (cis-pyran) and galactosylhydroxylysine were reduced in RA production and normalized after ASP-2pb intervention, and these eight metabolites were negatively correlated with RA symptoms such as posterior claw volume and plasma inflammatory cytokines including IL-1β, TNF-α, and IL-6, and are thought to be the key to the alleviation of RA by ASP-2pb intervention metabolites (23).

The most important among the metabolites associated with intestinal flora are short-chain fatty acids (SCFAs). On the one hand, SCFAs were mentioned in three of these seven studies, and on the other hand, SCFAs have an important regulatory role in RA (34). We have listed SCFAs-related studies separately (**Table 3**), which contains two studies in 2023 (18, 19) and one study in 2024 (21). Liu et al. measured SCFA levels in cecal contents and found that LBP treatment significantly increased propionic and butyric acid concentrations. These results suggest that LBP intervention modulates SCFA production through microbial fermentation, which subsequently reduces serum inflammatory factors and ultimately alleviates RA symptoms (18). Ye et al. demonstrated that NIP treatment increased the relative abundance of *Eisenbergiella*, a bacterial genus known for its butyrate-producing capacity (19). The 2024 study confirmed that ESP therapy elevated butyric acid levels, while revealing an inverse correlation between butyric acid and Enterococcus/Enterorhabdus abundance (21). These findings collectively emphasize the central role of SCFAs in mediating the therapeutic effects of TCMPs on RA through gut microbiota modulation.

#### 3.3.6 Regulatory effects of TCMPs on genes and proteins in gut microbiota-associated pathways

Among the nine included studies, we further summarized the pathways through which TCMPs exert therapeutic effects on RA and systematically cataloged the involved genes and proteins (**Table 3**). Four studies specifically elucidated potential mechanisms linking gut microbiota modulation to therapeutic outcomes (15–17, 21). Hu et al. demonstrated that Angelica sinensis polysaccharide (ASP) treatment upregulated Adcy4 (involved in calcium signaling), Cldn5 and Myl9 (tight junction-associated), as well as Slit3 and Rgs18 (bone remodeling-related genes) (15). Notably, bacterial taxa including *norank_f_norank_o_Clostridia_UCG-014*, *Candidatus_Saccharimonas*, and *norank_f_norank_o_RF39* showed positive correlations with these genes, while *norank_f_Oscillospiraceae* and *norank_f_Desulfovibrionaceae* exhibited negative correlations (15). Lai et al. delineated a methylation-dependent mechanism wherein LBP intervention reduced *Lachnospiraceae_NK4A136_group* and *uncultured_bacterium_f_Ruminococcaceae* while increasing *Romboutsia*, *Lactobacillus*, *Dubosiella* and *Faecalibaculum* - microbial shifts potentially elevating the methyl donor SAM. This consequently enhanced methylation of specific colonic epithelial genes (Dpep3, Gstm6, Slc27a2, Col11a2, Sycp2, SNORA22, Tnni1, Gpnmb, Mypn and Acsl6) and suppressed their expression (16). Liu et al. proposed a downstream mechanism based on the increase of the gut microbiota metabolite GGC by ASPS, which was able to interfere with the activation of NLRP3 inflammasome through inhibition of ASC nucleation and decrease the expression of caspase-1 and IL-1β (17). Ma et al. speculated that bacterial communities may act through secondary metabolites on the TLR4/HDAC/NF-κB signaling pathway (21). These findings collectively establish multi-tiered mechanisms whereby TCMPs modulate host gene/protein expression through microbiota-derived signals across immunological, epigenetic and metabolic pathways.

#### 3.3.7 Correlation between gut microbiota and biochemical indicators

Based on gut microbiota with reported relative abundance changes in at least two studies, we systematically analyzed their associations with RA-related parameters, inflammatory cytokines, bone quality, and SCFAs. Eleven of the sixteen examined microbial genera demonstrated significant associations with these clinical indicators. RA-related parameters included paw swelling and arthritis score; inflammatory markers comprised IL-1α, IL-1β, IL-12, IL-17A, IL-10, TNF-α, and IL-6; bone quality indices involved BV/TV, Tb.Th, BMD, BS/BV, BV, BV/TV, and SMI; while SCFAs measured were acetic acid, propionic acid, isobutyric acid, butyric acid, isovaleric acid, and valeric acid.

**Figure 5:**
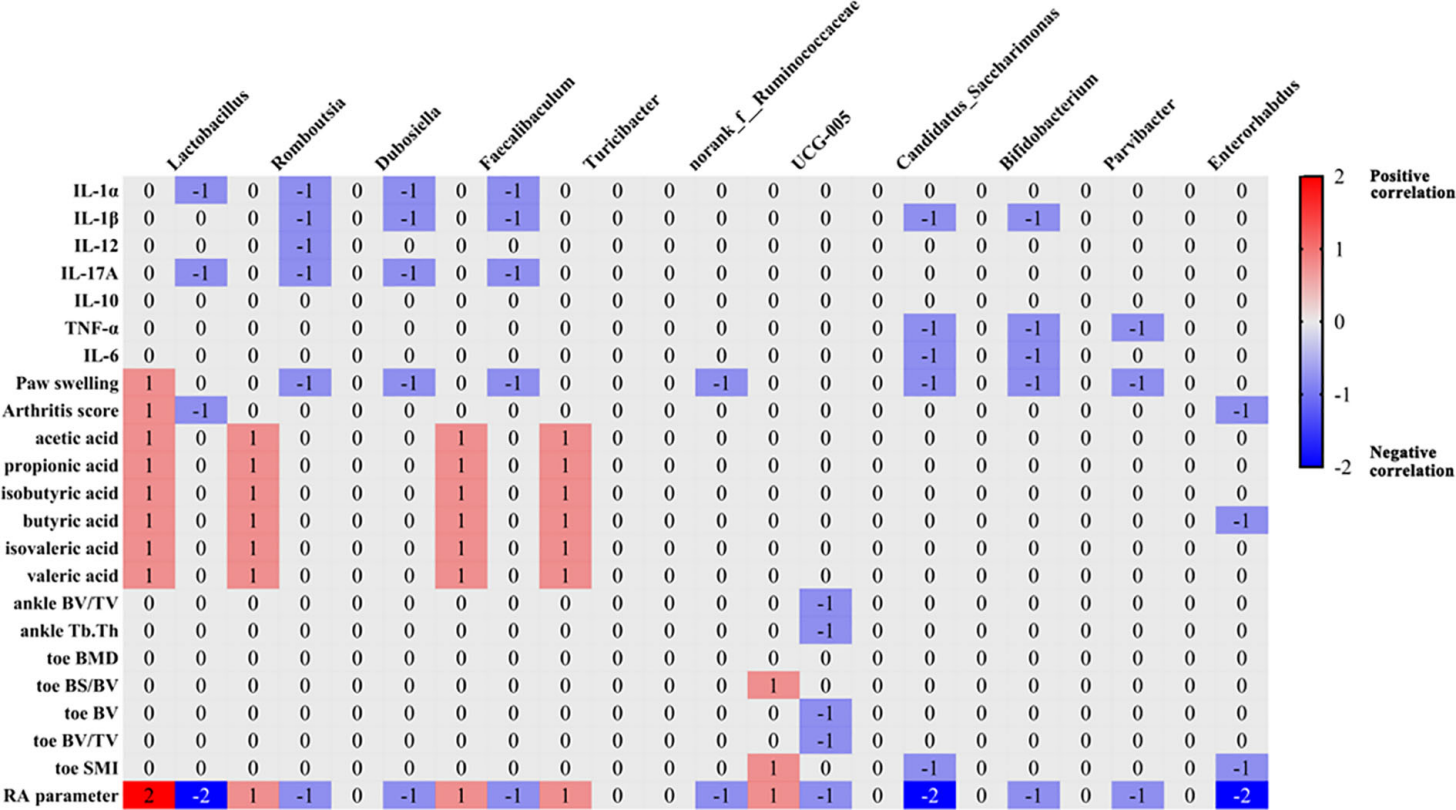
Summary of the correlation between intestinal bacteria and biochemical indexes. Numbers in the figure represent the number of articles. These articles refer to the analysis of the correlation between intestinal bacteria with changes after TCMPs intervention and RA parameters.

*Lactobacillus* emerged as the most frequently studied genus (4 studies) regarding its association with RA biochemical markers (15, 18, 19, 23), though findings showed some inconsistency. Most studies indicated negative correlations between *Lactobacillus* abundance and pro-inflammatory cytokines (16), alongside positive correlations with SCFA levels (18), collectively suggesting its protective role against RA progression. The 2025 study reinforced this by demonstrating negative correlations between *Lactobacillus* and paw swelling (23), aligning with previous conclusions. However, the opposite conclusion was suggested in another study, which concluded that *Lactobacillus* was positively associated with paw swelling and arthritis scores (20). Four additional genera — *Romboutsia*, *Faecalibaculum*, *Candidatus_Saccharimonas*, and *Enterorhabdus* — were investigated in two studies each. *Romboutsia* was positively correlated with short-chain fatty acids (18) and negatively correlated with inflammatory factors and paw swelling (16). *Faecalibaculum* was in agreement with *Romboutsia* results. *Candidatus_Saccharimonas* showed inverse relationships with paw swelling, inflammatory factors, and SMI. These consistent findings position these three genera as potentially beneficial microbiota for RA amelioration. *Enterorhabdus* demonstrated negative correlations with arthritis scores, SMI, and propanoic acid (20, 21). Notably, while a 2023 study reported decreased propanoic acid levels in RA models (18), the study examining the *Enterorhabdus*-propanoic acid relationship observed increased propanoic acid levels in RA models (21). This suggests that an elevation in *Enterorhabdus* abundance may reduce propanoic acid levels, thereby potentially alleviating RA severity (**Figure 5**).

Our integrative analysis identifies *Lactobacillus*, *Romboutsia*, *Faecalibaculum*, *Candidatus_Saccharimonas*, and *Enterorhabdus* as key microbiota significantly associated with RA biochemical profiles, with increased abundance generally correlating with symptom alleviation. Our investigation revealed that TCMP treatment significantly increased the abundance of *Lactobacillus* and *Faecalibaculum*. Furthermore, two separate studies consistently reported elevated levels of *Romboutsia* and *Candidatus_Saccharimonas* after polysaccharide intervention. Interestingly, we observed an unexpected significant decrease in *Enterorhabdus* after TCMP administration, contrary to our initial hypothesis. This paradoxical finding suggests that TCMPs may not exert their therapeutic effects on RA through modulation of this particular bacterial genus.

## 4 Discussion

This systematic review is the first to comprehensively evaluate the effects of TCMPs on RA through gut microbiota modulation in animal models. Analyzing nine studies that met the inclusion criteria, we found that TCMPs not only significantly alleviated the clinical symptoms and bone destruction indices of RA but also profoundly influenced the structure and metabolic function of the gut microbiota. These effects were primarily mediated by key microbial metabolites, particularly SCFAs.

The nine included studies consistently demonstrated that TCMPs significantly ameliorate disease activity and pathological damage in RA animal models. Specifically, TCMPs reduced arthritis scores, alleviated paw swelling, and improved bone microstructural parameters. The protective effects on articular cartilage and bone were validated across multiple studies. For instance, Ganoderma lucidum polysaccharides (GLP) have been shown to inhibit matrix metalloproteinase production and promote osteoblast formation (35). Similarly, Dendrobium huoshanense stem polysaccharide (cDHPS) reduced osteoclast differentiation, effectively alleviating cartilage erosion and bone destruction in CIA mice (36). Furthermore, TCMPs downregulated pro-inflammatory cytokine levels, upregulated anti-inflammatory cytokine expression, and inhibited the activation of key inflammatory signaling pathways. These findings align with previous reports on the anti-inflammatory and immunomodulatory effects of TCMPs. A novel polysaccharide (LJCP-2b) was isolated from Lonicerae Japonicae Caulis, and *in vitro* experiments demonstrated that LJCP-2b affects the function of TNF-α-induced rheumatoid arthritis fibroblast-like synoviocytes (RA-FLS), leading to decreased levels of IL-6 and IL-1β (37). Anoectochilus roxburghii polysaccharides (ARP) significantly inhibited the activation of the NF-κB pathway by suppressing the phosphorylation of I-κB and p65, thereby downregulating the mRNA expression of IL-1β and IL-6 in LPS-stimulated RAW 264.7 cells (8). Treatment with Astragalus polysaccharides also significantly reduced IL-1β-induced production of the pro-inflammatory cytokines IL-6 and TNF-α in fibroblast-like synoviocytes (9). The inhibitory effect of saposhnikovia divaricate polysaccharide (SDP) on RA rat fibroblast-like synoviocytes *in vitro* was similarly reflected in the suppression of TNF-α and IL-1β secretion (38). An arabinogalactan derived from *Cynanchum atratum* significantly reduced CIA severity and rebalanced the Treg/Th17 ratio, enhancing its immunosuppressive activity (39). GLP regulates the proliferation and differentiation of antigen-presenting cells such as dendritic cells and inhibits the phagocytosis of mononuclear macrophages and natural killer cells (35). GLP also modulates humoral and cellular immunity, including immunoglobulin production, T and B cell proliferation responses, and cytokine release (35). These studies further suggest that TCMPs exert therapeutic effects in RA through multi-target and multi-pathway mechanisms. Notably, some studies also found that TCMPs reduce oxidative stress levels. In an RA animal model, Thladiantha dubia fruit crude polysaccharide (TF-P) was shown to decrease MDA and nitric oxide (NO) levels while increasing SOD levels (40). Poria cocos polysaccharides (PCP) reduced reactive oxygen species (ROS) and MDA levels and increased antioxidant levels such as SOD and GSH (41). This suggests that antioxidant effects may also represent an important mechanism underlying their therapeutic action in RA.

Gut microbiota dysbiosis is widely reported in the pathogenesis of RA (33, 42–44). This review found that TCMPs significantly remodeled the gut microbiota structure in RA animal models. Regarding α-diversity, the effects of TCMPs were somewhat heterogeneous: some studies showed a significant increase, while others found no significant changes, which may be related to factors such as the animal model, polysaccharide type, and intervention duration. In contrast, β-diversity was significantly altered in most studies, suggesting that TCMPs have a relatively clear impact on the overall microbial community structure. At the phylum level, TCMPs primarily affected nine bacterial phyla, among which Patescibacteria, Firmicutes, Actinobacteria, and Bacteroidota were the most studied. Although some variability existed across studies, the overall trend indicated that TCMPs may reduce the F/B ratio, which is inversely correlated with the elevated F/B ratio reported in RA patients (45). At the genus level, TCMPs induced abundance changes in 65 bacterial genera. Beneficial bacteria such as *Dubosiella*, *Faecalibaculum*, and *Bifidobacterium* were consistently reported to increase across multiple studies, while *Enterorhabdus* was consistently decreased. This suggests that TCMPs may treat RA by increasing probiotics and suppressing harmful bacteria.

On one hand, TCMPs can act as prebiotics, providing nutritional substrates for beneficial gut bacteria (46). Their complex glycan chains, rich in various monosaccharides such as glucose, galactose, and mannose (47–49), can be utilized by beneficial bacteria like *Bifidobacterium* and *Lactobacillus*. These sugars are taken up via sugar transporters and participate in metabolism, promoting the proliferation of beneficial bacteria. Studies have shown that Dendrobium officinale polysaccharides (DOPs) increase the abundance of gut microbiota such as lactic acid bacteria and *Bifidobacterium* (50). In mouse experiments, 16S rRNA gene sequencing data revealed that PCP alone stimulated the growth of probiotics (e.g., *Lactobacillus*, *Bifidobacterium*) (51). Additionally, TCMPs significantly promote the growth of beneficial bacteria by regulating the intestinal environment. One study proposed that Bletilla striata polysaccharides (BP) significantly lower pH during *in vitro* fermentation (52), creating an acidic environment conducive to the growth of acidophilic beneficial bacteria such as *Bifidobacterium* and *Lactobacillus*, further promoting their proliferation and optimizing the gut microbiota structure, thereby contributing to the stability of the intestinal microecosystem.

Regarding the inhibition of harmful bacteria by TCMPs, several mechanisms have been proposed. First, inhibition can occur through disruption of bacterial cell membrane integrity. Research suggests that litchi pericarp polysaccharide (LPPs) can alter the cell wall integrity and membrane permeability of *Staphylococcus aureus*, leading to the leakage of intracellular macromolecules and small molecules, and inhibiting cell respiration and oxidative metabolism (53). Second, inhibition can occur through interference with key metabolic pathways. Experiments have found that Tetrastigma hemsleyanum Diels et Gilg’s polysaccharide (TP) can inhibit *Escherichia coli* proliferation by interfering with glycolysis and gluconeogenesis (54). In summary, TCMPs can inhibit the growth of harmful bacteria while indirectly promoting the growth of beneficial bacteria by modulating the structure and function of the gut microbiota (55).

Microbial metabolites, especially SCFAs, play a crucial regulatory role in the pathogenesis and treatment of RA (34, 56, 57). This regulation is highly dependent on changes in the abundance of SCFA-producing bacteria. For example, the abundance of the anti-inflammatory bacteria *Faecalibacterium prausnitzii* and *Roseburia* is significantly reduced in RA patients (58); both are major butyrate producers, and their deficiency leads to decreased local and systemic anti-inflammatory capacity (59). In this review, seven studies investigated the effects of TCMPs on microbial metabolites, with three specifically focusing on SCFAs. The results showed that TCMPs significantly increased levels of butyrate, propionate, and other SCFAs, which were closely correlated with reduced inflammatory cytokines and improved RA symptoms. TCMPs intervention was shown to promote SCFA production by modulating the gut microbiota (60, 61). Mechanistically, SCFAs exert anti-RA effects by multi-dimensionally regulating immune cell function. At the T cell level, SCFAs promote Treg differentiation, inhibit Th17 cell polarization, and reduce the release of pro-inflammatory cytokines such as IL-1β, IL-6, and TNF-α (57, 62, 63). Another study also suggested that butyrate inhibits the expression of inflammatory factors like IL-1β, IL-6, and IL-17A in a CIA mouse model, potentially by inducing CD4+ T cell differentiation into Treg cells and promoting anti-inflammatory IL-10 production, thereby regulating Th17 cell function (64). At the B cell level, SCFAs can modulate B cell subset distribution; administering SCFAs before CIA onset in mice increased the frequency of Bregs and reduced the proportion of transitional and follicular B cells (65). Furthermore, the role of SCFAs extends beyond immune cell regulation to include protection via enhancement of intestinal barrier integrity. One of the included studies indicated that ESP exerted anti-RA effects by increasing SCFA levels such as butyrate, which in turn enhanced intestinal barrier integrity (upregulating ZO-1 expression, repairing intestinal mucosal damage) (19). Thus, SCFAs can upregulate the expression of tight junction proteins, thereby maintaining intestinal barrier function and preventing bacterial translocation and systemic immune activation. This mechanism synergizes with immunomodulatory effects to constitute the integrated effect of SCFAs in treating RA. For example, butyrate can alleviate intestinal barrier damage by targeting zonulin and tight junction proteins while simultaneously inhibiting osteoclasts and autoantibody production, balancing systemic T and B lymphocyte immune responses, thereby synergistically suppressing bone erosion and inflammation (66).

Beyond SCFAs, TCMPs also modulate various other microbiota-related metabolites, such as SAM, GGC, amino acid derivatives, and lipid metabolites. These metabolites may mediate the anti-inflammatory and immunomodulatory effects of TCMPs through pathways such as DNA methylation (16) and NLRP3 inflammasome inhibition (17). This finding suggests that the regulation of microbial metabolism by TCMPs is diverse and complex, and future research should further explore the functional significance of non-SCFA metabolites.

Some studies have further elucidated the molecular mechanisms by which TCMPs regulate host gene expression and immune responses through the microbiota and its metabolites. For instance, ASP was shown to upregulate tight junction-related genes (*Cldn5*, *Myl9*) and bone remodeling-related genes (*Slit3*, *Rgs18*), and these genes were positively correlated with specific bacterial taxa such as *Candidatus_Saccharimonas* (15). LBP may alter gut microbiota composition, increasing the level of the methyl donor SAM, thereby inducing methylation and suppressing expression of specific genes in colonic epithelial cells (16). Additionally, ASPS was found to increase the metabolite GGC, which inhibits ASC nucleation, blocks NLRP3 inflammasome activation, and consequently reduces caspase-1 and IL-1β levels (17). These mechanisms collectively construct a multi-level “microbiota-metabolite-host” regulatory network, providing an important basis for understanding the complexity of TCMPs in treating RA.

Through correlation analysis of genera with reported relative abundance changes in at least two studies, we identified *Lactobacillus*, *Romboutsia*, *Faecalibaculum*, *Candidatus_Saccharimonas*, and *Enterorhabdus* as the genera most closely associated with RA biomarkers. Among these, increased Lactobacillus abundance correlated with decreased pro-inflammatory cytokines, increased SCFAs, and reduced paw swelling. Although one study reported a contrary finding, the overall trend supports its protective role. Clinically, increased intestinal *Lactobacillus* is also suggested to be beneficial for alleviating RA (67). Increased *Romboutsia* and *Faecalibaculum* correlated with increased SCFAs and decreased inflammatory cytokines, suggesting they are potentially beneficial bacteria. This inference is supported by drug intervention studies showing that increasing the abundance of these two genera can restore intestinal microecological balance and exert anti-RA effects (68). However, the role of certain genera in RA remains controversial across studies. For example, this review found that the abundance of *Candidatus_Saccharimonas* was negatively correlated with paw swelling, inflammatory cytokine levels, and SMI, suggesting a potential beneficial role. Conversely, another study observed that *Candidatus_Saccharimonas* abundance was significantly increased in the gut of CIA rats and positively correlated with pro-inflammatory cytokines, joint swelling, and pathological scores; its abundance decreased after drug intervention, coinciding with RA symptom relief (69). Similarly, this review found that *Enterorhabdus* was significantly decreased after TCMP treatment, yet it showed a negative correlation with arthritis scores, SMI, and propionic acid levels. These seemingly contradictory observations suggest that the role of different bacterial genera in RA may be species- or functional-subtype-specific, and future studies should integrate metagenomics and strain-level analysis to clarify these complexities.

**Figure 6:**
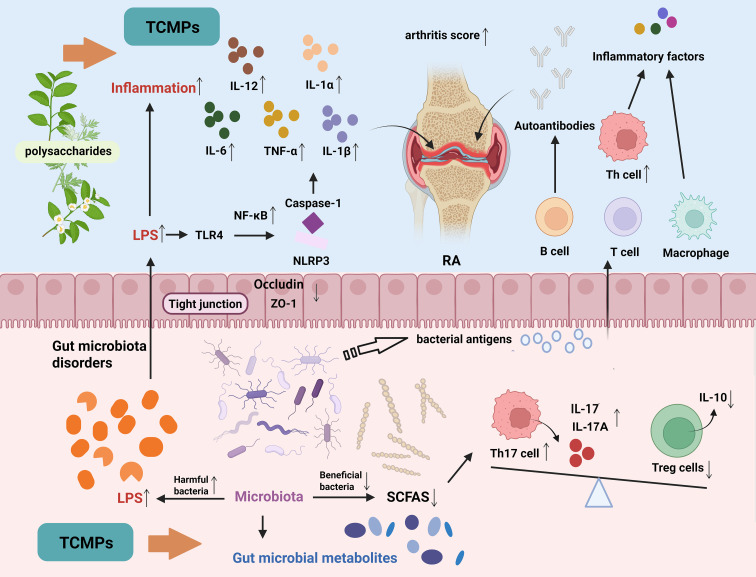
Mechanism of TCMPs in regulating intestinal microecology to alleviate RA.

In summary, TCMPs exert anti-RA effects through multi-target regulation of gut microbiota structure, microbial metabolites, and host immune responses, forming a multi-level “microbiota-metabolite-host” regulatory network. The overall mechanism is illustrated in **Figure 6**.

This review assessed the quality of included studies using the SYRCLE Risk of Bias tool. The results showed that all studies had total scores between 2 and 3 (out of a maximum of 10), indicating low overall methodological quality. Major issues included unclear randomization methods, lack of description of allocation concealment, absence of blinding during experiment implementation and outcome assessment, and incomplete reporting of animal numbers and data in some studies. These deficiencies may introduce selection, performance, and detection biases, thus affecting the reliability of the results. Although all studies reported positive effects of TCMPs on RA and gut microbiota, these conclusions should be interpreted with caution due to the generally low methodological quality.

Furthermore, this review has the following limitations: First, the included studies were all animal experiments, and caution is needed when extrapolating the findings to clinical settings. Second, significant heterogeneity existed across studies regarding the source, extraction method, molecular weight, monosaccharide composition, and dosing regimen (dose, route, intervention duration) of the polysaccharides, increasing the heterogeneity of the results. Third, some studies did not include a positive control group, limiting the comparability of therapeutic efficacy. Fourth, most microbiota analyses employed 16S rRNA sequencing, which offers limited resolution and cannot reveal changes at the species level or in functional genes. Fifth, only a few studies conducted systematic analyses of metabolites, and the functions of metabolites other than SCFAs remain unclear.

Based on the findings and limitations of this review, future research should focus on optimizing study design by strictly implementing randomization, controlled conditions, and blinding to improve the methodological quality of animal studies, while standardizing the reporting of animal numbers and data completeness. Mechanistic research should be deepened by integrating multi-omics approaches such as metagenomics, metabolomics, and transcriptomics to systematically elucidate the structure-activity relationships of TCMPs and their “microbiota-metabolite-immunity” regulatory networks. Analysis should advance from the genus level to the species and functional gene levels to identify key effector strains and their metabolic pathways. Clinical translation should be promoted through rigorously designed clinical trials to verify the efficacy and safety of TCMPs in RA patients and explore their synergistic effects with conventional drugs. Finally, given the crucial role of polysaccharide structure in their activity, establishing comprehensive quality standards covering source, extraction, purification, and characterization is essential to ensure comparability and reproducibility of research results.

## 5 Conclusion

This systematic review demonstrates that TCMPs exert significant anti-RA effects in animal models by modulating gut microbiota structure and its metabolites, particularly SCFAs. The underlying mechanisms involve enriching beneficial bacteria, suppressing pathogenic bacteria, enhancing intestinal barrier function, regulating immune cell differentiation and balance, and mediating systemic anti-inflammatory and bone-protective effects through microbial metabolites. Although current evidence supports the potential of TCMPs as therapeutic candidates for RA, the conclusions should be interpreted with caution due to generally low study quality and high heterogeneity. Future research should prioritize high-quality, multi-omics integrated basic research and clinical translational studies to provide a more solid scientific basis for the precise treatment of RA with TCMPs through gut microbiota modulation.

## Data Availability

The original contributions presented in the study are included in the article/**Supplementary Material**. Further inquiries can be directed to the corresponding authors.

## Glossary

16S rDNA: 16S ribosomal DNA
16S rRNA: 16S ribosomal RNA
AGs: arabinogalactans
AIA: adjuvant-induced arthritis
ASC: apoptosis-associated speck-like protein containing a CARD
ASP: Angelica sinensis polysaccharide
ASPS: Acanthopanax senticosus polysaccharide
BMD: bone mineral density
BP: Bletilla striata polysaccharides
BS: bone surface
BV: bone volume
BV/TV: bone volume/total volume
cDHPS: Dendrobium huoshanense stem polysaccharide
CFA: complete Freund’s adjuvant
CIA: collagen-induced arthritis
DBZP: Dianbaizhu polysaccharide
DOPs: Dendrobium officinale polysaccharides
ESP: Ephedra sinica polysaccharide
F/B ratio: Firmicutes/Bacteroidota ratio
GGC: γ-glutamylcysteine
GLP: Ganoderma lucidum polysaccharides
GSH: glutathione
HDAC: histone deacetylase
HWE: hot water extraction
IL-1α: interleukin-1 alpha
IL-1β: interleukin-1 beta
IL-6: interleukin-6
IL-10: interleukin-10
IL-12: interleukin-12
IL-17: interleukin-17
IL-17A: interleukin-17A
JAK/STAT3: Janus kinase/signal transducer and activator of transcription 3
LBP: Lycium barbarum polysaccharide
LJCP-2b: Lonicerae Japonicae Caulis polysaccharide 2b
LPS: lipopolysaccharide
LPPs: litchi pericarp polysaccharides
MAE: microwave-assisted extraction
MDA: malondialdehyde
MyD88: myeloid differentiation primary response 88
NF-κB: nuclear factor-kappa B
NIP: Notopterygium incisum polysaccharide
NLRP3: NLR family pyrin domain containing 3
PCP: Poria cocos polysaccharides
PLE: pressurized liquid extraction
PRISMA: Preferred Reporting Items for Systematic Reviews and Meta-Analyses
PROSPERO: International Prospective Register of Systematic Reviews
PS: polysaccharide
RA: rheumatoid arthritis
RA-FLS: rheumatoid arthritis fibroblast-like synoviocytes
RCT: randomized controlled trial
ROS: reactive oxygen species
SAM: S-adenosylmethionine
SCFAs: short-chain fatty acids
SDP: Saposhnikovia divaricata polysaccharide
SMI: structure model index
SOD: superoxide dismutase
SYRCLE’s RoB tool: Systematic Review Centre for Laboratory Animal Experimentation’s Risk of Bias tool
Tb.N: trabecular number
Tb.Th: trabecular thickness
TCM: traditional Chinese medicine
TCMPs: traditional Chinese medicine polysaccharides
TF-P: Thladiantha dubia fruit crude polysaccharide
Th17: T helper 17 cell
TLR4: Toll-like receptor 4
TNF-α: tumor necrosis factor-alpha
TP: Tetrastigma hemsleyanum polysaccharides
TRAF6: TNF receptor associated factor 6
Treg: regulatory T cell
TV: total volume
UAE: ultrasound-assisted extraction
WTD: Wu-tou decoction.
